# P-361. Outcome of Candida auris Point Prevalence Survey in a Tertiary Care Hospital in Southeast Michigan

**DOI:** 10.1093/ofid/ofae631.562

**Published:** 2025-01-29

**Authors:** Ambreen Malik, Anita Shallal, Abigail Ruby, Jessica Malm, Jennifer Mclenon, Clare Shanahan, Eman Chami, George J Alangaden, Geehan Suleyman

**Affiliations:** Henry Ford Hospital, Detroit, Michigan; Henry Ford Health, Detroit, Michigan; Henry Ford Health, Detroit, Michigan; Henry Ford Healt, Detroit, Michigan; Henry Ford Health, Detroit, Michigan; Henry Ford health, Detroit, Michigan; Henry Ford Hospital, Detroit, Michigan; Henry Ford Health, Detroit, Michigan; Henry Ford Health, Detroit, Michigan

## Abstract

**Background:**

*C. auris,* an emerging multi-drug resistant organism associated with nosocomial outbreaks, is becoming more prevalent in Southeast Michigan due to interfacility healthcare transfer. The CDC recommends contact isolation and screening of epi-linked healthcare contacts of newly identified *C. auris* patients to prevent spread and assess potential transmission. We describe our institution’s experience with *C. auris* point prevalence surveys (PPS).

**Figure:**
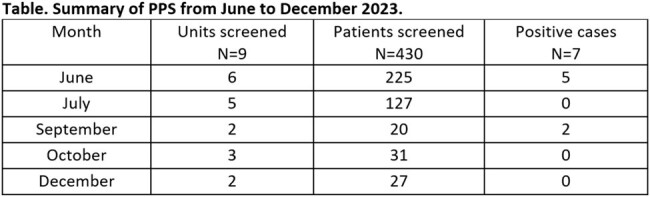

**Methods:**

PPS was conducted at an 877-bed academic, tertiary care center consisting of private and semi-private rooms in Detroit. In collaboration with Michigan Department of Health and Human Services, we performed PPS by swabbing the axilla and groin of all patients on the implicated unit when a *C. auris* index case was incidentally identified from June-Dec 2023. In addition, potentially exposed roommates were flagged in the electronic health record for future screening. When additional cases were identified, 2 negative PPS conducted biweekly were required to stop PPS.

**Results:**

Six *C. auris* index cases were identified during the 6-month period. We performed 18 PPS on 9 units and screened 430 patients (Table); 5 refused and 14 were discharged prior to screening. Four (22%) of the PPS resulted in identification of new cases; 2 PPS yielded 1 case each, 1 PPS yielded 3 cases, and the final PPS yielded 2 cases. Of those screened, 7 (1.6%) tested positive on 4 units. Nosocomial transmission was implicated in 4 patients across 2 units after an epidemiologic investigation. Terminal cleaning was performed on units implicated in nosocomial transmission, in addition to ancillary areas visited by the affected patients. Infection Prevention emphasized the importance of adhering to hand hygiene, appropriate PPE use, and cleaning and disinfection of the environment and shared equipment.

**Conclusion:**

Controlling the spread of *C. auris* is a public health priority. However, only 1.6% of cases were detected with intense PPS. Despite considerable effort and healthcare resources being utilized to identify additional cases and mitigate possible nosocomial transmission, *C. auris* continues to spread in our region. Alternatively, healthcare facilities should consider active surveillance of high-risk patients at the point of hospitalization to mitigate the need for PPS.

**Disclosures:**

**All Authors**: No reported disclosures

